# Supplementary material for: Inflammatory, Oxidative Stress, and Apoptosis Effects in Zebrafish Larvae after Rapid Exposure to a Commercial Glyphosate Formulation
Source: Biomedicines. 2021 Nov 27;9(12):1784. doi: 10.3390/biomedicines9121784 (PMC8698920; doi:10.3390/biomedicines9121784)
Supplement: Supplementary file 1 [file biomedicines-09-01784-s001.zip › biomedicines-1468040-supplementary.pdf]

Table S1 – Original values of biochemical parameters evaluated in zebrafish WT larvae with 72 hpf, exposed for 4:30 h to different chemicals

Biochemical parameters	Groups					Statistical test	*p*
			RF (µg a.i. mL^{-1})				
	Control (E3)	$CuSO_4$ 2.5 µg mL^{-1}	1	5	10		
**ROS**	**196 (178-204)[a]**	**272 (239-275)[c]**	**221 (206-232)[ab]**	**214 (191-247)[ab]**	**246 (237-265)[bc]**	**X^2 (4) = 17.130**	**0.02**
**SOD**	**10 ± 1.3[abc]**	**8.8 ± 2.4[ab]**	**8.5 ± 1.1[a]**	**12 ± 1.5[c]**	**11.7 ± 1.2[bc]**	**F (4. 20) = 6.317**	**0.002**
CAT	4.49 ± 0.8	5 ± 0.4	3.2 ± 1.1	5.1 ± 1	3.9 ± 1	F (4. 20) = 4.646	0.08
GPx	2.6 ± 0.7	1.8 ± 0.5	3.2 ± 0.8	2.1 ± 1	1.8 ± 0.5	F (4. 20) = 1.374	0.279
**GSH**	**103 ± 9.9[ab]**	**117 ± 18.1[b]**	**94 ± 11.7[b]**	**89 ± 10.4[a]**	**96 ± 4.9[ab]**	**F (4. 20) = 3.894**	**0.017**
GSSG	668 ± 205	612 ± 43	606 ± 160	575 ± 179	675 ± 66.4	F (4. 20) = 0.584	0.678
**OSI**	**0.164 ± 0.038[ab]**	**0.19 ± 0.018[b]**	**0.165 ± 0.045[b]**	**0.164 ± 0.044[ab]**	**0.1443 ± 0.013[a]**	**F (4. 20) = 4.426**	**0.01**
GST	13.3 ± 3.3	13.8 ± 2.6	15 ± 2.5	11.2 ± 2.9	10.1 ± 1.7	F (4. 20) = 2.292	0.095
**LPO**	**2.51 ± 0.43[ab]**	**1.85 ± 0.36[a]**	**2.31 ± 0.67[ab]**	**2.83 ± 0.61[b]**	**2.44 ± 0.62[ab]**	**F (4. 20) = 2.889**	**0.049**
LDH	57.62 ± 3.1	64.2 ± 10	58.72 ± 3.6	59.32 ± 8.1	59.94 ± 5.2	F (4. 20) = 0.193	0.940
AChE	17 ± 4.39	13 ± 2.8	17.5 ± 4	15.3 ± 4.1	11.5 ± 2.1	F (4. 20) = 1.528	0.232
**NO**	**5.87 ± 3.3[a]**	**16.3 ± 3[b]**	**4.3 ± 3.4[a]**	**7.1 ± 2.7[ab]**	**9 ± 5.6[ab]**	**F (4. 15) = 4.317**	**0.016**

Data from at least five independent replicates of 50 animals each, is expressed as mean ± SD for parametric data distribution or median (25th–75th quartile) for non-parametric data. Statistical analysis was performed using one-way ANOVA followed by Tukey's multiple-comparison test or Kruskal-Wallis followed by Dunn's test. Different letters indicate significant statistical differences between groups ($p < 0.05$).
